# Supplementary material for: Functionnectome as a framework to analyse the contribution of brain circuits to fMRI
Source: Commun Biol. 2021 Sep 2;4:1035. doi: 10.1038/s42003-021-02530-2 (PMC8413369; doi:10.1038/s42003-021-02530-2)
Supplement: Supplementary file 2 — Supplementary Information [file 42003_2021_2530_MOESM2_ESM.pdf]

SUPPLEMENTARY INFORMATION

Functionnectome as a framework to analyse the contribution of brain circuits to fMRI

Victor Nozais[1,2*], Stephanie J. Forkel[1,2,3], Chris Foulon[4], Laurent Petit[1], Michel Thiebaut de Schotten[1,2*]

[1]*Groupe d'Imagerie Neurofonctionnelle, Institut des Maladies Neurodégénératives-*UMR 5293, CNRS, CEA, *University of Bordeaux, Bordeaux, France*

[2]*Brain Connectivity and Behaviour Laboratory, Sorbonne Universities, Paris, France*

[3]*Centre for Neuroimaging Sciences, Department of Neuroimaging, Institute of Psychiatry, Psychology and Neuroscience, King's College London, London, UK*

[4]*Institute of Neurology,* UCL, *London* WC1N 3BG, UK

**Corresponding authors:* victor.nozais@gmail.com ; michel.thiebaut@gmail.com

Supplementary note 1: Region-wise analysis

The Functionnectome has the ability to project the functional signal of each brain voxel onto the white matter. Although the principle is relatively straightforward, the computation can require up to several hundred thousand steps per subject, depending on how many voxels are kept for the analysis. This is quite computationally intensive and thus requires powerful computers and servers, which are not necessarily available to all. In order to make the Functionnectome accessible to the broadest audience, we added the possibility to use brain regions instead of voxels as spatial units, reducing the number of processing steps to a few hundred per subject. This simplification of the analyses decreases the computation time from hours per subject (or even days on consumer-grade computers) to minutes. The brain regions used for this example of simplified analysis, and the associated probability maps, were derived from the HCP multi-modal parcellation (MMP) and are included in the priors provided with the software. During the analysis, the functional signal from a given region at a specific time-point is defined as the median of the values of that region's voxels at that time-point. Here, we used the median instead of the mean in order to mitigate the effect of noisy voxels in small regions.

All the analyses presented in the main body of the article were replicated using the region-wise analysis aforementioned. For the sake of comparison, we kept the same activation-values threshold as in the original analyses presented in the main text.

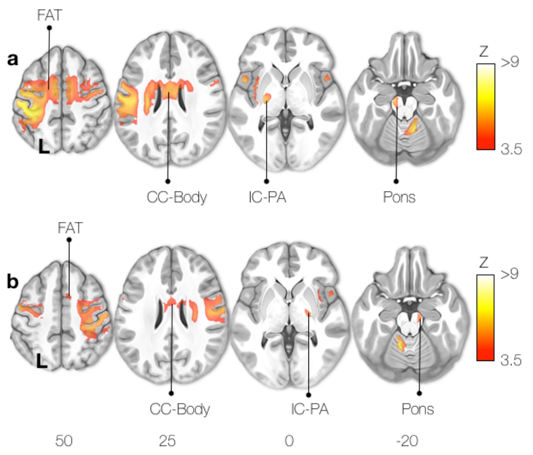

Supplementary Figure 1: Right (a) and left (b) finger-tapping motor activation network from the region-wise Functionnectome analysis. FAT: Frontal Aslant Tract; CC-Body: Corpus Callosum body; IC-PA: Internal capsule posterior arm.

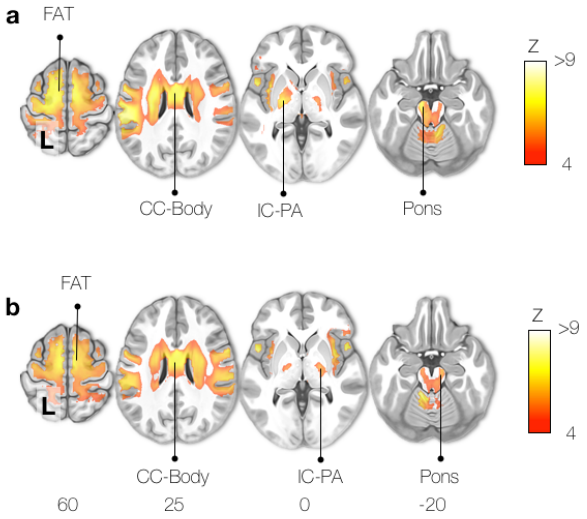

Supplementary Figure 2: Motor task activation network for right (a) and left (b) toes clenching, from the region-wise Functionnectome analysis. FAT: Frontal Aslant Tract; CC-Body: Corpus Callosum body; Pons; IC-PA: Internal capsule posterior arm.

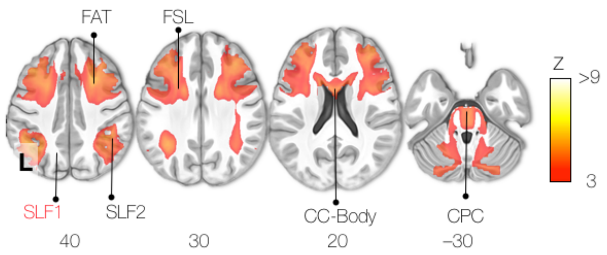

Supplementary Figure 3: Working memory task activation network from the region-wise Functionnectome analysis. Pathways not present here but appearing in the voxel-wise analysis are labelled in red. FAT: Frontal Aslant Tract; FSL: Frontal Superior Longitudinal tract; SLF: Superior Longitudinal Fasciculus; CC-Body: Corpus Callosum body. CPC: Cortico-Ponto-Cerebellar tract. Tracts indicated in red were identified in the original voxel-wise analysis but missing in the simplified region-wise analysis.

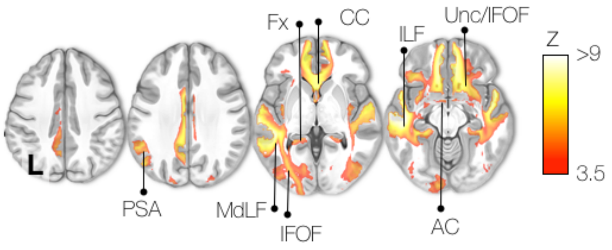

Supplementary Figure 4: Semantic system task activation network from the region-wise Functionnectome analysis. Pathways appearing in the voxel-wise analysis but not present here are labelled in red. PSA: Posterior Segment of the arcuate fasciculus; MdLF: Middle Longitudinal Fasciculus; IFOF: Inferior Fronto-Occipital Fasciculus; Fx: Fornix; CC: Corpus Callosum; ILF: Inferior Longitudinal Fasciculus; Unc: Uncinate fasciculus; AC: Anterior Commissure.

Supplementary discussion 1

The region-wise analysis delivers results comparable to those obtained with the voxel-wise analysis, albeit slightly less sensitive and specific. For instance, compared to the voxel-wise analysis, a few pathways were missing in the region-wise analysis (e.g. the first branch of the superior longitudinal fasciculus - SLF1 - in the working memory task map - Supplementary figure 3), and other tracts were

displaying larger activations (e.g. the left uncinate/IFOF in the semantic system task map - Supplementary figure 4). The choice of brain segmentation used for the simplified region-wise analysis could improve the results. Here, the multimodal parcellation of the HCP has a general-purpose, but using a brain parcellation tailored for the specific analysis of a project could yield better results.

Overall, the region-wise analysis offers a way for any research team to use the Functionnectome and obtain results in a short amount of time, independently from the computational resources available. Even teams considering the application of the voxel-wise analysis could use it as a quick test of their method, before investing the time and computational resources for the complete analysis.

Supplementary note 2: Functionnectome User-guide

Version 0.1.1

Content

Introduction *7*

Disclaimer 7

Definitions 8

Requirements (Read this before starting!) 8

Overview 9

Comprehensive guide *10*

Installation 10

Loading the input 4D volume 10

Voxel-wise and region-wise analysis 13

Number of processors to use 14

White matter anatomical priors 14

Output options 15

Save, Load, Launch & Exit 16

Frequently Asked Questions and advanced functions *17*

How long will the software take to process py data? 17

How do I use the Functionnectome with command lines only? 17

Can I manually change/create a setting file? 18

How do I specify different paths to alternate priors? 18

How do I make my own anatomical priors? 19

How do I create my own HDF5 file for my priors? 19

How do I use the Functionnectome on a distant computational grid/server? 20

Will this work on my OS? 20

Will parallelizing the analysis use more RAM? 20

I think you are doing something wrong / poorly optimized… 20

Introduction

The Functionnectome is a Python-based tool to allow projecting functional signals (or more generally grey matter information) onto the white matter. As input, the software accepts **NifTI 4D volumes (usually *f*MRI files)**, uses predefined (but modifiable if needed) white matter **priors** for the computation, and **outputs functionnectome 4D volumes**. The user can choose between navigation through commands or a GUI (Graphical User Interface).

The generated functionnectomes 4D volumes can then be processed with the same statistical tools used to analyze fMRI. For example, the functionnectome generated from a classic task fMRI can be analyzed with the usual tools (e.g. generalized linear model) to reveal the white-matter pathways involved with the task.

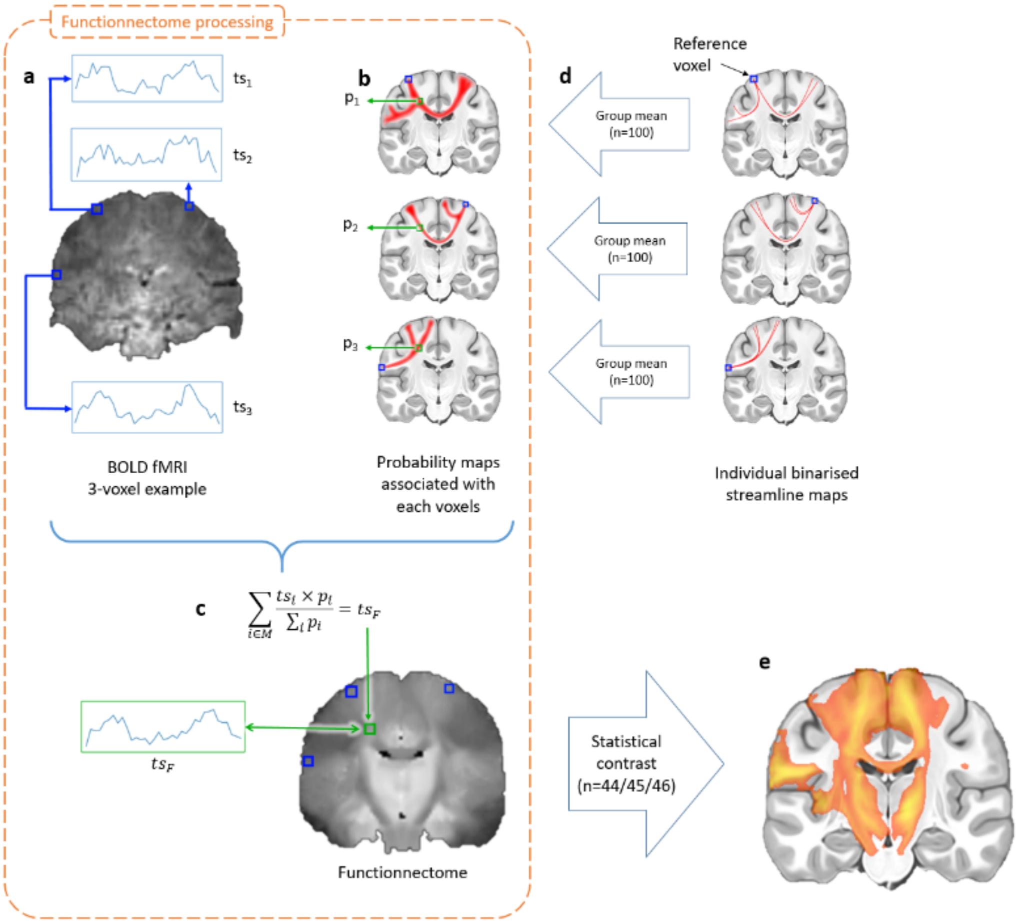

Disclaimer

No financial conflicts.

Not licensed for medical use.

The software and its codes are licenced under the GNU General Public License.

Definitions

- "Functionnectome" (with an uppercase "F") refers to the overall method and the program applying the method. "functionnectome" (with a lowercase "f") refers to the 4D volume resulting from the application of the Functionnectome.
- "Voxel-wise" analysis refers to the projection of functional signals onto the white matter as this is done at the voxel level. "Region-wise" analysis runs on predefined parcels of brain regions, grouping voxels together and reducing the number of steps to the order of hundreds at most, though at the cost of precision.

Requirements (Read this before starting!)

1. The Functionnectome runs on **Python 3.6** (or higher). In addition to the basic Python distributions, four python libraries are needed: **Numpy, Pandas, Nibabel** and **H5py**. If you install the Functionnectome toolbox using pip (see the "Installation" section), they will be installed automatically. Otherwise, you will have to install them manually on your python virtual environment.
2. Depending on the size of the input data, RAM usage can vary. An 8-12GB RAM is recommended, but for small input volumes, 4GB might suffice.
3. Expected input files are **NifTI (.nii or .nii.gz) 4D volumes in MNI152 space, with 2mm^3 isotropic voxels**.
4. Input files should either be:
    - All in the **same folder,** e.g. :
        - "*/myHome/**dataFMRI**/subject01.nii*"
        - "*/myHome/**dataFMRI**/subject02.nii*"
    - Or in **different folders but with "similar paths,"** i.e., with the same number of subfolders and the folder with the data ID at the same depth, e.g. :
        - "*/myHome/**subject01**/data/mydata.nii*"
        - "*/myHome/**subject02**/data/mydata.nii*"
5. For a voxel-wise analysis, a brain (or rather gray-matter) **mask in MNI152 space, with 2mm^3 isotropic voxels,** selecting the voxels with "interesting" functional signal, is also necessary. The mask should be a **NifTI 3D volume**, with the same spatial dimensions as the main 4D volume input.
6. The **white matter anatomical priors** are available online (https://www.dropbox.com/s/22vix4krs2zgtnt/functionnectome_7TpriorsH5.zip?dl=0), but the toolbox gives you also the option to download them automatically.
7. The provided priors require input data to be in the **MNI152 space, with 2mm^3 isotropic voxels.**
8. We strongly recommend running the Functionnectome on a machine equipped with multiple CPUs (8 cores or more). At least for the voxel-wise analysis. The region-wise analysis is much less computationally demanding. Note that process parallelisation should not increase the RAM load.

Overview

In the GUI, the analysis parameters can be set and the input and the output can be defined. The Functionnectome analysis can be launched from the GUI once the parameters are set. The settings can be saved and loaded for future analysis.

- Input: 4D volume, NifTI
- (opt.) Input mask: 3D volume, NifTI. (required for voxel-wise analysis)
- Output1: the functionnectome, 4D volume, NifTI
    - The user defines the general output folder
    - A subfolder is generated for voxel-wise or region-wise analysis
    - A second subfolder is generated with the unique ID* of the input data
    - The output 4D data is saved there and named "functionnectome.nii.gz"
    - Example: "/myHome/myOutput/voxelwise_analysis/subject01/functionnectome.nii.gz"
- Output2: 3D volume, NifTI.
    - sum_ probaMaps_voxel.nii.gz or sum_ probaMaps_region.nii.gz
    - Stored in the same directory as Output1 (for voxel-wise analysis) or in the general output folder (for region-wise analysis)
    - Sum of all probability maps used for a given subject (depends on the mask)
    - Used during the processing, might be useful for alternative processing

*Unique ID: If all input files are in the same folder, the program uses the filenames (without the extension). If the files are in different folders with similar paths (cf. bullet 4 in ***Requirements***), the ID is the name of the folder (e.g. it will be "subject01" *in the example given above with "/myHome/**subject01**/data/mydata.nii*")

A quick guide to run an analysis:

1. Select the input 4D files by clicking on either "Choose files" buttons (depending on your data storage organisation).
2. Select whether you want a region-wise or voxel-wise analysis
    - If you chose the voxel-wise approach, select the masks that will filter out unwanted voxels. You can either choose one mask per input file or choose one unique mask applied to all input files.
3. Select the number of parallel processes you want to launch concurrently. The more processors, the faster the analysis. Importantly, the priors should be saved on a storage device with fast reading speed. For an estimation of the time needed to run the Functionnectome on your data, check the FAQ at the end of the manual.
4. Choose the file type of the priors. If the priors of the software are to be used they are made available as HDF5 files. Where users wish to use their own templates we recommend using the same file type.
5. If the priors have not yet been downloaded (or the path leading to the files is not set), you will be offered to automatically download it (or asked to select the file path). This should only happen once, when you first try to run the Functionnectome.
6. Choose the output folder. If the provided path doesn't exist, it will be automatically generated.
7. Choose whether or not you want to mask the output to remove voxels outside of the brain (as defined by a brain template). We recommend it (default behaviour).
8. Launch the analysis. Or save the settings in order to launch it later or with a command line.

Comprehensive guide

Installation

The project can be download through pip using the command line:

- pip install git+https://github.com/NotaCS/Functionnectome.git
  or
- python -m pip install git+https://github.com/NotaCS/Functionnectome.git

You can also manually download the project from GitHub:

https://github.com/NotaCS/Functionnectome

The priors will be automatically downloaded during the first launch of the Functionnectome, but you can also download them manually here:

https://www.dropbox.com/s/22vix4krs2zgtnt/functionnectome_7TpriorsH5.zip?dl=0

If you download the priors manually, you will need to unzip the file. The path to the priors will be asked by the program during the first run.

Loading the input 4D volume

Once you have downloaded the package, launch the GUI. To do so, there are 3 possible ways:

- **If the toolbox was installed using pip**, simply call "FunctionnectomeGUI" (without the quotation marks) in a command window using the virtual environment where you installed the Functionnectome.
- **If you manually downloaded the codes**, run the functionnectome_GUI.py script from a python interpreter, or run "python functionnectome_GUI.py" in a command window.

In the GUI, you can specify the input data (assumed to be BOLD data, but any 4D nifty files are accepted). Two ways of selecting the files are possible depending on how they are stored on your computer:

1. ALL FILES IN 1 FOLDER: If they are all in the same folder, click on the "Choose files" button just under the "Select BOLD files from one folder" label.

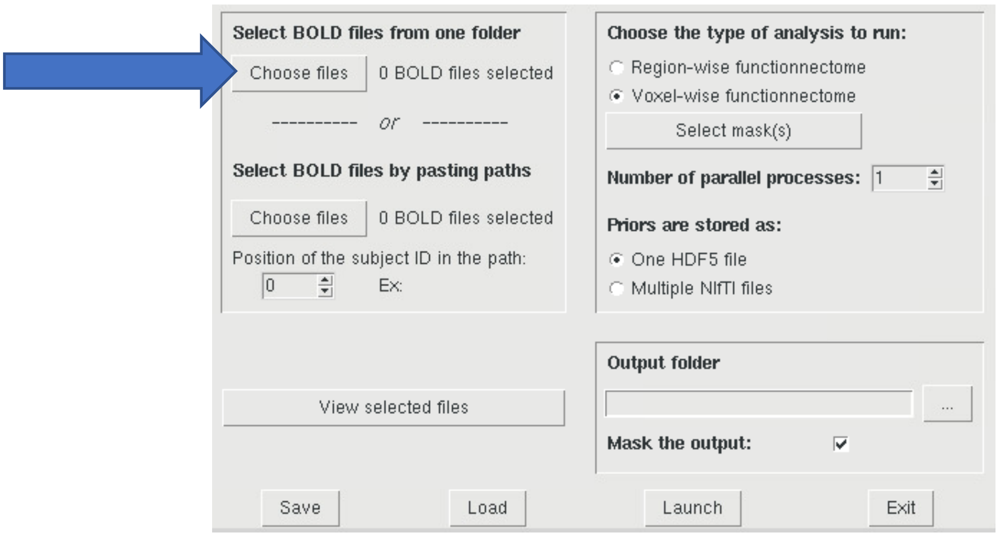

A generic interactive window will open to allow you to choose the files from a folder. You can manually select the files you want to keep/discard. Only NifTI files are accepted (extension ".nii" or ".nii.gz").

2. SEPERATE FOLDERS FOR EACH PARTICIPANT: If the files are in different folders (but properly organised with folders and sub-folders following the same pattern), click on the "Choose files" button under the "Select BOLD files by pasting paths" label.

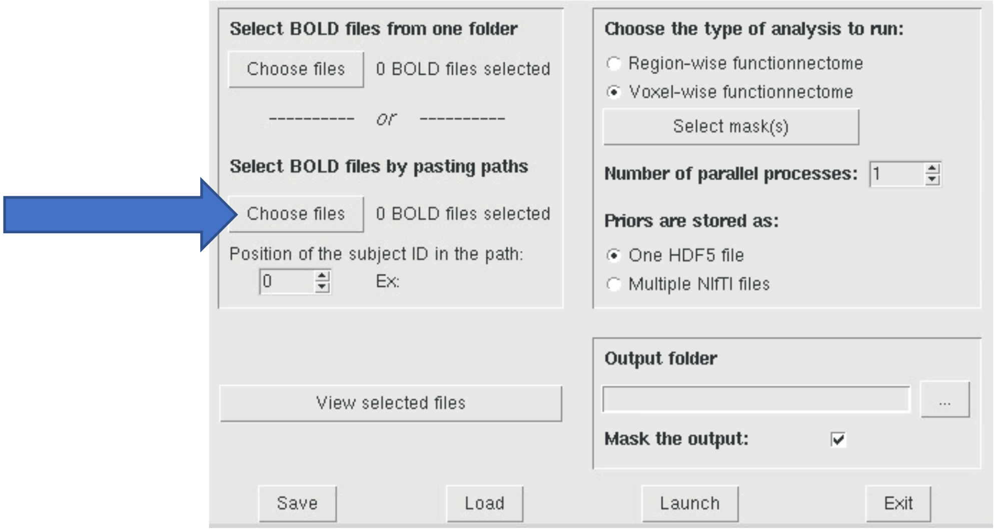

A new window will open. In the central box, you can paste the paths of each file. An easy way to obtain the paths of all the files requires the use of a command window (i.e. a terminal):

- **On MacOS/Unix/Linux**, use the "ls" command. For example, let's say that all my files are stored in paths as: "/myhome/current_project/subject????/functionalFiles/fMRI_acquisition.nii.gz"
  with "????" being a unique ID (e.g a number) that is different for every subject. Use as many "?" as there are digits in the ID numbers. To display all the file-paths, simply enter the command (in the terminal): "**ls -1** /myhome/current_project/subject????/funFiles/fMRI_acquisition.nii.gz"
  Here, "ls" will list all the paths, and the "-1" option will display one path per line.
- **On Windows**, use the "dir" command, similarly to the above-mentioned "ls". For example: "dir C:\myHome\project\subject????\fMRI_acquisition.nii.gz"

Copy the displayed result and paste it into the GUI.

If for some reason you enter or paste something wrong in the GUI text box, you can easily correct it manually or clear the whole box by clicking on "Clear". When you are done, click on "OK" to accept the file-paths. Or "Cancel" to return to the main window without keeping the paths or the changes made to existing paths.

Advanced tip: Once you have files selected, you can manually edit them (add/remove) by opening the "pasting" window and adding ore removing a line, even if you selected the all from one folder. Note that the added paths should still keep the same naming pattern as the other already there. Likewise, if you "Clear" the window and click on "OK", it will delete every path (not the files, of course, just the paths stored by the GUI). If you accidentally modify or clear the paths, simply click on "Cancel".

To differentiate the input files and to name the output folders, a unique ID is attributed to each of them. When selecting all inputs from the same folder (option 1), the unique ID is the name of the file (without the extension). When selecting from multiple similar folders (option 2), the program will automatically select the first divergence in the paths as the ID. The position of the ID in the path is displayed, and an example of ID (from the first selected path) is given. Subsequently, the GUI gives you the option to manually change the location in the paths where the program finds the IDs, using the little arrows near the position number:

List of the paths given:

/myHome/workspace/myProject/myAcquisition/MRI/subject01/fMRI/readyData.nii.gz
/myHome/workspace/myProject/myAcquisition/MRI/subject02/fMRI/readyData.nii.gz
/myHome/workspace/myProject/myAcquisition/MRI/subject03/fMRI/readyData.nii.gz

Position of the subject ID in the path (index of the folder in the path, **starting at "0"**):
/myHome/workspace/myProject/myAcquisition/MRI/subject01/fMRI/readyData.nii.gz

Here, "[illegible] 0 ct01" ha 1 en autor 2 ally selec 3 s it is t 4 st (a 5 nly i 6 examp 7 vergence between the paths.

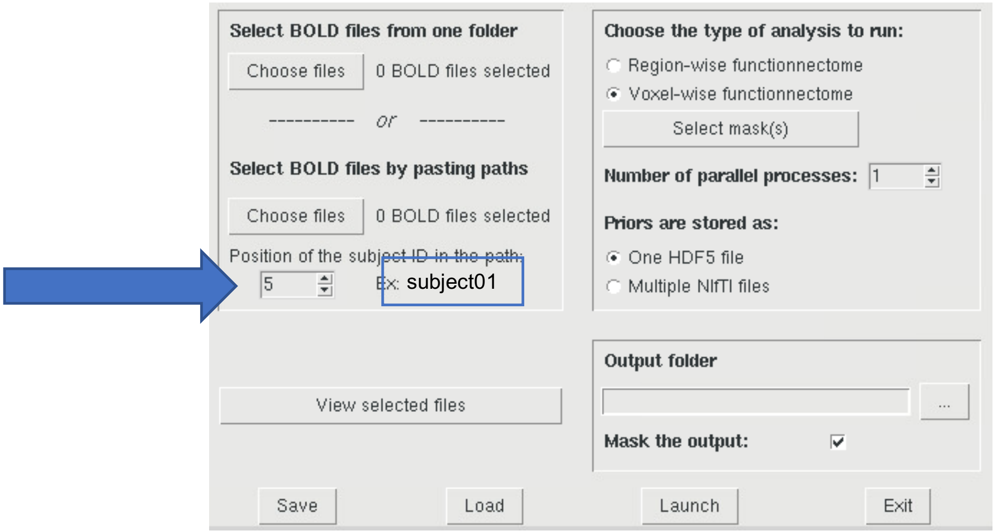

Sometimes, the first divergence is not the one you want to keep. You can then manually modify the position. For example, if we have the paths:

/myHome/myProject/session1/subject01/readyData.nii.gz

/myHome/myProject/session1/subject02/readyData.nii.gz

/myHome/myProject/session2/subject03/readyData.nii.gz

/myHome/myProject/session2/subject04/readyData.nii.gz

The number of the session is the first divergence between the paths, so the position will be automatically set at "2", and the example given will be "session1". The user will then have to change the position to "3" (which will change the example to "subject01").

Voxel-wise and region-wise analysis

Select whether you want to run a voxel-wise or a region-wise analysis. If you have enough computational power, we recommend using the voxel-wise analysis.

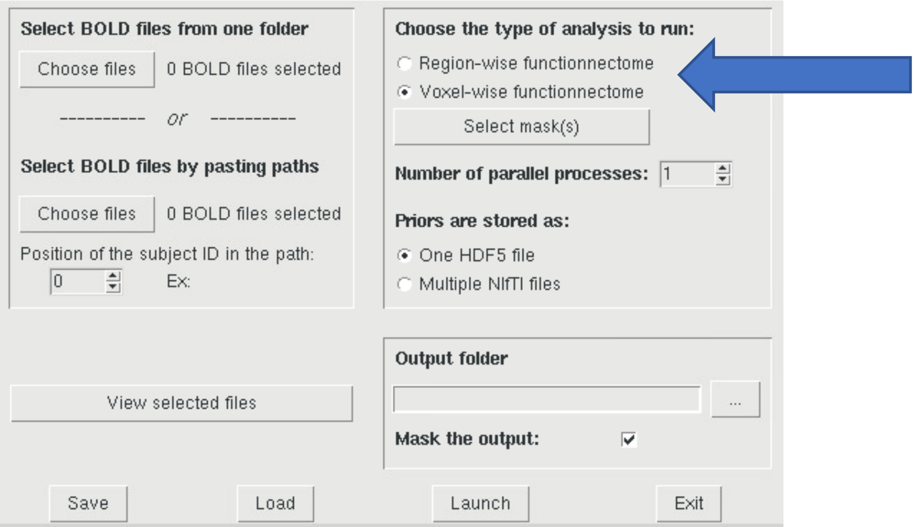

If you select the voxel-wise analysis (i.e. "voxel-wise functionnectome"), you will need to specify the mask(s) that will filter out unwanted voxels. Click on "Select mask(s)". A new window will pop open:

You can either attribute one mask per input file ("One mask per subject"), usually useful if the

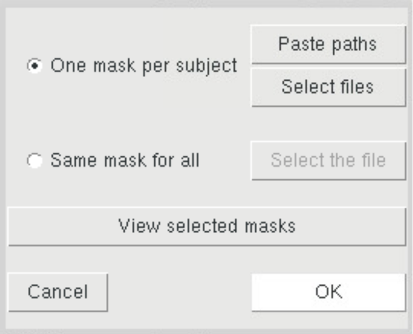

masks are created depending on the individual data; or select one unique mask that will be similarly applied to all inputs, for example, a mask delimiting the grey matter.

Selecting one mask per input uses the same procedure as the selection of the input 4D volumes (Manual selection in one folder, or pasting the paths).

The paths of **the 4D inputs and the masks** are automatically and systematically **sorted**. Consequently, these **inputs and the masks** must have the **same naming convention** because the pairing between a mask and an input 4D volume is done using the position of the paths in the corresponding list. Using the "View selected files" and "View selected masks" buttons, check the paths to verify that this is correct.

The region-wise analysis uses priors derived from a brain parcellation. In the provided default priors, we used the HCP multi-modal parcellation (MMP, doi.org/10.1038/nature18933). The functional signal of a region is defined as the median of the voxel values at a given time point.

Number of processors to use

The Functionnectome processing time can be significantly cut down by parallelizing it. You can choose the number of processes (i.e. the number of CPU or core used at the same time). The maximum number of parallel processes is automatically detected and set as a top limit. The parallelization happens at the subject level, so only one subject is run at a time, which limits the RAM load.
The Functionnectome parallel processing system does not support the spread of the

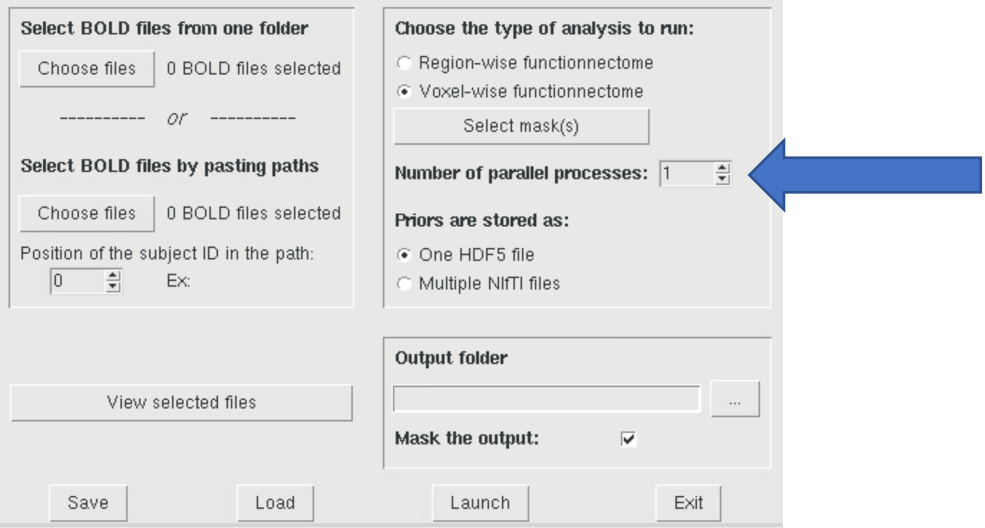

computation across multiple machines/nodes. All the CPUs must be mounted on the same machine.
As an example, using 16 processes in parallel, the processing time of a typical fMRI scan with the voxel-wise analysis can be around 4 hours (but it depends a lot on the size of the input and how many voxels are kept with the mask).

White matter anatomical priors

As the Functionectome projects BOLD onto the brain's connections, the priors are necessary. They consist of the white matter probability maps and a brain template. The template is a T1 brain template and is used to define the dimensions of the images and to delimit the brain. The program can access them from two different types of storage:

- A collection of NifTI files, with a folder containing all the probability maps (voxel-wise or region-wise), and a brain template (same dimensions as the probability maps). This option might be better if you need to easily change (some of) the priors.
- An HDF5 priors file (containing all the required images). This format allows for rapid access to the files, fast transfer of the file from one location to another, and because it is not easy to change, it reduces the risk of accidentally deleting necessarily data. Check the FAQ "*How do I specify different paths to alternate priors?*" if you want to create your own priors.

Paths to the priors are saved in a file and are purposely not readily available (to encourage traceability practices). Ideally, the paths to the priors should be set once (when downloading them) and left alone. It is possible to specify alternative paths directly in the settings file (see FAQ "*How do I specify different paths to alternate priors?*"). For ease of use and to reduce the margin for errors, the settings file is always saved with the results. This way you'll always know what priors you used (default or manually changed).

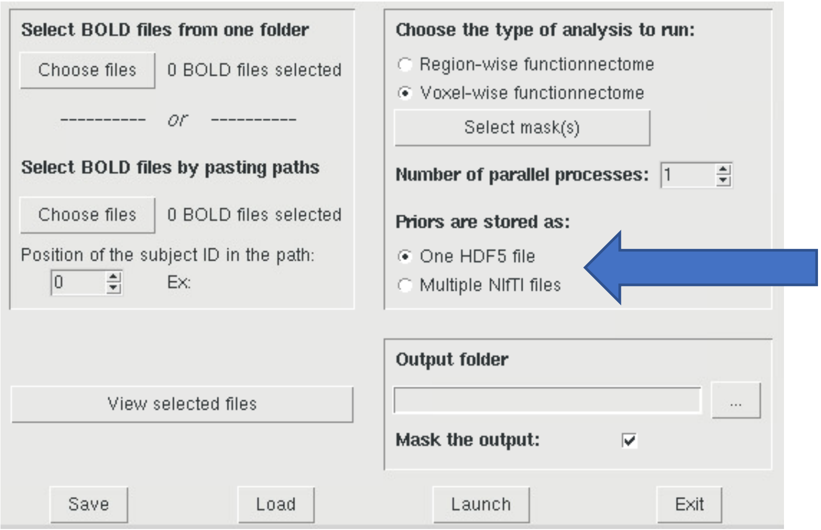

Output options

Choose the output folder by either clicking on the "..." button or by copy-and-pasting the path to the text-box.

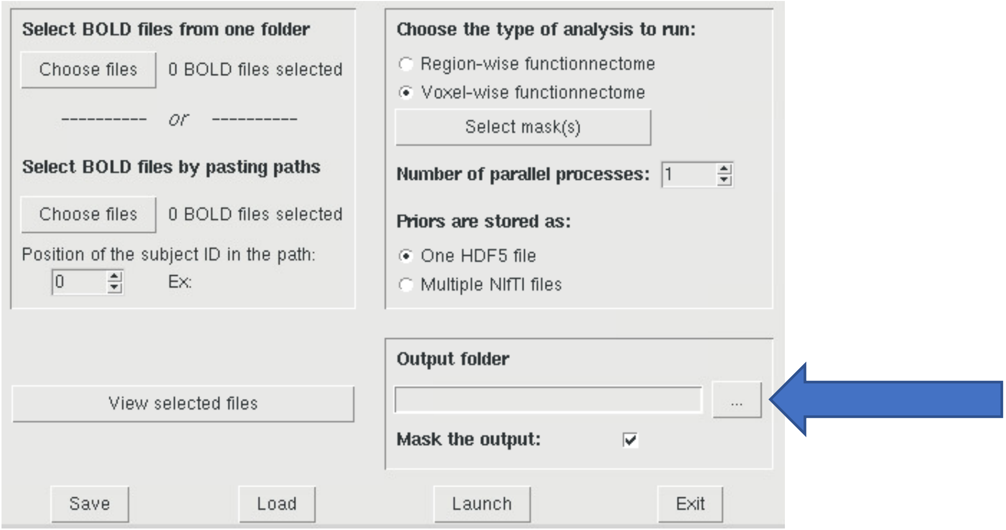

If the folder does not exist, it will be automatically created. It will serve as a "root" folder for the analysis, where the settings file (resulting from the parameters you entered in the GUI) and the additional sub-folders will be stored. Multiple analyses can use the same output folder, as long as the IDs of the input data are different.

Typically, if you select "/myHome/myProject/myOutputFolder/" as the path to the output folder, the results will be stored in this structure (assuming you are doing a voxel-wise analysis):

/myHome/myProject/myOutputFolder/

- settings.fcntm (text file containing the analysis parameters)
- voxelwise_analysis/
  - subject01/
    - functionnectome.nii.gz
    - sum_probaMaps_voxel.nii.gz
  - subject02/
    - functionnectome.nii.gz
    - sum_probaMaps_voxel.nii.gz
  - subject03/
    - functionnectome.nii.gz
    - sum_probaMaps_voxel.nii.gz
  - subject04/
    - functionnectome.nii.gz
    - sum_probaMaps_voxel.nii.gz

The last parameter available is the "Mask the output" option. If checked (recommended and set as default), the functionnectome will be masked to remove all voxels outside of the brain (as defined by the brain template, which is part of the priors).

Save, Load, Launch & Exit

The buttons at the bottom of the GUI:

- Save: Saves the parameters entered on the GUI in a simple text file (with a "*.fcntm*" extension). It can be opened with any text editor. This settings file can be used to manually launch the Functionnectome from a terminal. All the parameters must be properly entered in the GUI before saving is possible.
- Load: Loads a settings file ("*.fcntm*") to fill the GUI parameters. Useful if you want to run a different analysis using the same input 4D files.
- Launch: Saves the parameters in the output folder, closes the GUI, and runs the analysis using the provided settings.
- Exit: Closes the GUI without doing anything.

Frequently Asked Questions and advanced functions

How long will the software take to process my data?

It really depends on a lot of factors. The size of the input data, but also the type of processors, the type of storing device on which the priors are written, and the number of processes all play a part.

On our machines, we estimated a value of 0.004 sec/voxel/volume. So, to get an estimation of the computation time:

Duration (sec.) = 0.004 x $Voxel_{Mask}$ x NbVol / NbCore

- $Voxel_{Mask}$: Number of voxels in the mask used in voxel-wise analysis. Replace by the number of regions in region-wise analysis.

- NbVol: Number of volumes (i.e. time point) in your input 4D file.

- NbCore: Number of cores (CPU) used to run the computation.

For example, running the Functionnectome in voxel-wise analysis, using a gray-matter mask containing 180,000 voxels, and with an fMRI scan of 1,050 volumes, on a machine with 16 cores, gives:

- 0.004 x 180,000 x 1,050 / 16 = 47,250 sec ≈ 13 hours

Using the region-wise analysis drastically cuts the time needed, even using a less powerful machine (**at the cost of some precision and sensitivity in the final results**). Indeed, with the same input fMRI volume, using a segmentation of 438 brain areas (as in the priors), with a 4-core machine, we get:

- 0.004 x 438 x 1,050 / 4 = 460 sec ≈ 8 min

Note that is approach will give you more of an order of magnitude than a precise result conserning the time needed for your processes. Importantly, some of our test seems to show that using more than 16 parallel processes doesn't not seem to improve the duration. This issue is being investigated.

How do I use the Functionnectome with command lines only?

You first need to create a settings file for your analysis (see below "Can I manually change/create a setting file?"). Let's assume it is called "settings.fcntm" and saved in /myPath/

Then:

- If you manually downloaded the scripts and saved it in, e.g. /myPath/, you can run the script by simply calling:
  python -u /myPath/Functionnectome/functionnectome.py /myPath/settings.fcntm
- If you installed the Functionnectome package using pip:
  - You can directly call "Functionnectome" in a command window (with the correct virtual environment) followed by the path to the settings file, e.g. "Functionnectome */myPath/settings.fcntm*".
  - Or you can find where the *functionnectome.py* script is stored and use it as is shown above.
  - You could also create a simple script importing the package, then running the function. For example, something like that:

```
from Functionnectome.functionnectome import run_functionnectome
fpath="/myPath/settings.fcntm"
run_functionnectome(fpath)
```

If you save it as "*/myPath/runFunctionnectome.py*", you can then simply call (in the correct python environment):
python -u /myPath/runFunctionnectome.py

Can I manually change/create a setting file?

Sure, it is a simple text file on which we changed the extension to ".fcntm". You can create your own file in a text editor (or copy a saved settings file from the GUI), you just need to make sure that the internal architecture is the same as in a GUI generated file.

Don't forget to have the "Number of subjects" and "Number of masks" values matching the respective numbers of paths you provide in the file.

The Functionnectome program possesses a simple system to check if the file is correctly organized, so if your custom settings file is completely off the mark, you'll get a notification.

Note that the "Position of the subjects ID in their path" starts at 0 (first folder). The last part of the path (i.e. the name of the file) can be designated with -1. This "-1" index is actually what is used when loading all the 4D input files from a single directory.

You can find examples of settings files in the project directory.

How do I specify different paths to alternate priors?

Since we strongly recommend not changing the default path to the priors too often (for the sake of traceability), we provide an alternative way to change the priors used for a given analysis. This can be especially useful when working on a machine different from usual, as is the case when running analyses on a computer grid/cluster or a distant server.
To give non-default priors or override the absence of default priors, copy and paste the following lines at the end of the settings file (with the ".fcntm" extension) you are using as input for the Functionnectome program (add an empty line before the first "###" in the file):

```
###
HDF5 path:
	/myHome/myProject/priors/functionnectome_priors.h5
Template path:
	/myHome/myProject/anat/brainT1template.nii.gz
Probability maps (voxel) path:
	/myHome/myProject/priors/probaMaps_voxels
Probability maps (region) path:
	/myHome/myProject/priors/probaMaps_atlas
Region masks path:
	/myHome/myProject/priors/decomposed_atlas
###
```

The labels, written in green above, must always be present. Conversely, you only need to fill in the paths (in blue above) that are useful. For example, if you are using an HDF5 priors file, you only need to enter the first path. The lines where the unnecessary path would have been written must be kept though, with empty lines:

```
###
HDF5 path:
	/myHome/myProject/priors/functionnectome_priors.h5
Template path:

Probability maps (voxel) path:
```

```
Probability maps (region) path:

Region masks path:

###
```

An example file using this option can be found in the project directory:

- settings_example2.fcntm

How do I make my own anatomical priors?

We do not have a readily available, easy to use code yet.

We used the "disconnectome" tool of the BCBtoolkit - http://toolkit.bcblab.com/ -, with one-voxel masks for the "lesions", for every brain voxel.
In essence, you are free to generate any kind of probability maps that reflect the probability of connection from each voxel to the rest of the brain, using the method of you choice. On that subject, we encourage you to build your own priors to better fit the type of analysis you are planning to do, or simply try and improve upon ours priors.
Note that the tractography used to generate the priors provided here used a deterministic approach, with parameters tuned to provide the most accurate results with regard to axonal tracing studies and with post-mortem dissections.

How do I create my own HDF5 file for my priors?

We provide a script to export the priors in NIfTI format into a single HDF5 file.
If you installed the package through pip, you can directly call "MakeH5" from a terminal. Otherwise, you can find the script in the project/package under the name makeHDF5prior.py. The command requires 5 arguments, each being the path to one of the input data. For example:

MakeH5 -o */myProject/myPriors.h5* -tpl */myData/brainT1template.nii.gz* -vxl */myData/myVoxelwisePriors* -reg */myData/myRegionwisePriors* -rmas */myData/myParcellation*

With:

- */myProject/myPriors.h5* the output path to the generated HDF5 file.
- */myData/brainT1template.nii.gz* the input path to the brain template file (used to mask the brain)
- */myData/myVoxelwisePriors* the input path to the folder containing the voxel-wise priors
- */myData/myRegionwisePriors* the input path to the folder containing the region-wise priors
- */myData/myParcellation* the input path to the folder containing the masks of the brain regions used to generate the region-wise priors

Both voxel-wise and region-wise priors must be given, but if you plan to only use one type of analysis, you can provide "fake" folders for the unwanted priors. These fake folders should then contain at least 1 NIfTI image with the proper geometry (for example, a copy of the template image should do), with a proper filename (see below).

The script will detect all the NifTI files in the given folders, and import them into the HDF5 file. The **voxel-wise probability maps** files **must** all be named with the following pattern: something_xx_yy_zz.nii.gz

"something" can be any string of character, but ***must not contain a "_".*** "xx", "yy", and "zz" are the spatial coordinates of the specific voxel linked to the given probability map. Be aware that these are the "raw" coordinates, before applying the transformation matrix (quaternion) given in the NifTI header.

The **region-wise probability maps** and their associated **region masks must** have the same name.

For example, the two probability maps

*/myData/myRegionwisePriors/*region1.nii.gz

and

*/myData/myRegionwisePriors/*region2.nii.gz

must be associated with the region masks

*/myData/myParcellation/*region1.nii.gz

and

*/myData/myParcellation/*region2.nii.gz

respectively.

(The paths can be absolute or relative paths, both are accepted by the script)

How do I use the Functionnectome on a distant computational grid/server?

The simplest way would be to transfer the functionnectome.py script and the priors (in the HDF5 file) to the machine you plan to use. Prepare manually the settings file (see "*Can I manually change / create a setting file?*") and give the location of the HDF5 prior on the distant machine as an alternate priors file (see "*How do I specify different paths to alternate priors?*"). Then run the script with the settings file as the argument (see "*How do I use the Functionnectome with command lines only?").*

Will this work on my OS?

The scripts have been tested on a Linux machine. It should work perfectly fine on a Mac. It hasn't been tested on Windows yet, but was coded with portability in mind, so it might work. Proper tests on different OS are planned.

Will parallelizing the analysis use more RAM?

No. Or at least at least it shouldn't. The script uses shared memory between processes to avoid RAM overload, and more processes should only split the data in smaller chunks, not copy it multiple times.

I think you are doing something wrong / poorly optimized…

If you have any advice, as long as the discussion remains respectful, we are happy to hear it. Leave a comment on the GitHub page or send us an e-mail.
