# Supplementary material for: Functionnectome as a framework to analyse the contribution of brain circuits to fMRI
Source: Commun Biol. 2021 Sep 2;4:1035. doi: 10.1038/s42003-021-02530-2 (PMC8413369; doi:10.1038/s42003-021-02530-2)
Supplement: Supplementary file 3 — Reporting Summary [file 42003_2021_2530_MOESM3_ESM.pdf]

nature research

Corresponding author(s): Victor Nozais & Michel Thiebaut de Schotten

Last updated by author(s): Jul 6, 2021

Reporting Summary

Nature Research wishes to improve the reproducibility of the work that we publish. This form provides structure for consistency and transparency in reporting. For further information on Nature Research policies, see our Editorial Policies and the Editorial Policy Checklist.

Statistics

For all statistical analyses, confirm that the following items are present in the figure legend, table legend, main text, or Methods section.

n/a	Confirmed	
☐	☒	The exact sample size (n) for each experimental group/condition, given as a discrete number and unit of measurement
☐	☒	A statement on whether measurements were taken from distinct samples or whether the same sample was measured repeatedly
☐	☒	The statistical test(s) used AND whether they are one- or two-sided<br>*Only common tests should be described solely by name; describe more complex techniques in the Methods section.*
☐	☒	A description of all covariates tested
☐	☒	A description of any assumptions or corrections, such as tests of normality and adjustment for multiple comparisons
☐	☒	A full description of the statistical parameters including central tendency (e.g. means) or other basic estimates (e.g. regression coefficient) AND variation (e.g. standard deviation) or associated estimates of uncertainty (e.g. confidence intervals)
☐	☒	For null hypothesis testing, the test statistic (e.g. F, t, r) with confidence intervals, effect sizes, degrees of freedom and P value noted<br>*Give P values as exact values whenever suitable.*
☒	☐	For Bayesian analysis, information on the choice of priors and Markov chain Monte Carlo settings
☒	☐	For hierarchical and complex designs, identification of the appropriate level for tests and full reporting of outcomes
☐	☒	Estimates of effect sizes (e.g. Cohen's d, Pearson's r), indicating how they were calculated

Our web collection on statistics for biologists contains articles on many of the points above.

Software and code

Policy information about availability of computer code

Data collection	Data from the publicly available Human Connectome Project database.
Data analysis	BCBtoolkit 4.1; Functionnectome 0.1.0; open software FSL 6.0, Python 3, NiBabel 3.1.1, scikit-learn 0.23, Tract Querier, Surf Ice 2 September 2019.<br>Code available with the manuscript https://github.com/NotaCS/Functionnectome

For manuscripts utilizing custom algorithms or software that are central to the research but not yet described in published literature, software must be made available to editors and reviewers. We strongly encourage code deposition in a community repository (e.g. GitHub). See the Nature Research guidelines for submitting code & software for further information.

Data

Policy information about availability of data

All manuscripts must include a data availability statement. This statement should provide the following information, where applicable:
- Accession codes, unique identifiers, or web links for publicly available datasets
- A list of figures that have associated raw data
- A description of any restrictions on data availability

The functionnectomes and the associated maps are available on demand to the authors. All the raw anatomical and functional data are available on the HCP website.
https://www.humanconnectome.org

Field-specific reporting

Please select the one below that is the best fit for your research. If you are not sure, read the appropriate sections before making your selection.

- [x] Life sciences
- [ ] Behavioural & social sciences
- [ ] Ecological, evolutionary & environmental sciences

For a reference copy of the document with all sections, see nature.com/documents/nr-reporting-summary-flat.pdf

Life sciences study design

All studies must disclose on these points even when the disclosure is negative.

Sample size	n = 44 for the motor tasks (left hand, right hand, left foot, right foot) n = 45 for the visual working memory task and n = 46 for the language (semantics) task. Task-fMRI with 40 participants is considered standard practice in the field of task-fMRI. Using more participants would not be representative and will not help demonstrate the Functionnectome methods/software's validity and interest. The number of participants corresponded to all the data available in the human connectome project replication dataset.
Data exclusions	There is no data exclusion. We included all data with test-retest in the human connectome project dataset.
Replication	For each analysis reported above, we repeated the analysis in a replication dataset of the same participants (same session, opposite phase of acquisition). Table 1 in the manuscript indicates the reproducibility rate of the results. The Functionnectome results were systematically more reproducible (r = 0.82 +/- 0.06) than the classical analysis (r = 0.72 +/- 0.05).
Randomization	No randomization was necessary for the design of our analysis. In the principal analysis, the same subjects were processed using the classical and the functionnectome analysis. For the replication analysis, the same subjects were measured twice with fMRI. The authors are happy to extend the analysis to different subjects if it is judged necessary.
Blinding	The study is data-driven; data are fully available for replication; therefore blinding was not relevant here.

Reporting for specific materials, systems and methods

We require information from authors about some types of materials, experimental systems and methods used in many studies. Here, indicate whether each material, system or method listed is relevant to your study. If you are not sure if a list item applies to your research, read the appropriate section before selecting a response.

Materials & experimental systems

n/a	Involved in the study	
x		Antibodies
x		Eukaryotic cell lines
x		Palaeontology and archaeology
x		Animals and other organisms
	x	Human research participants
x		Clinical data
x		Dual use research of concern

Methods

n/a	Involved in the study	
x		ChIP-seq
x		Flow cytometry
	x	MRI-based neuroimaging

Human research participants

Policy information about studies involving human research participants

Population characteristics	The 46 participants from the test-retest dataset of the Human Connectome Project. 67% Female, between 22 and 35 years old, healthy.
Recruitment	Full test-retest dataset from the Human Connectome Project.
Ethics oversight	Human Connectome Project

Note that full information on the approval of the study protocol must also be provided in the manuscript.

Magnetic resonance imaging

Experimental design

Design type	Task, block design
Design specifications	Each task design is fully described on the HCP website: https://protocols.humanconnectome.org/HCP/3T/task-fMRI-

protocol-details.html

Behavioral performance measures: As per the HCP procedure.

Acquisition

Imaging type(s): Functional

Field strength: 3T

Sequence & imaging parameters:
Sequence: Gradient-echo EPI
TR: 720 ms
TE: 33.1 ms
flip angle: 52 deg
FOV: 208x180 mm (RO x PE)
Matrix: 104x90 (RO x PE)
Slice thickness: 2.0 mm; 72 slices; 2.0 mm isotropic voxels
Multiband factor: 8
Echo spacing: 0.58 ms
BW: 2290 Hz/Px

Area of acquisition: Whole brain

Diffusion MRI ☒ Used ☐ Not used

Parameters: Diffusion data from 100 subjects of the 7T dataset were used in this study. From the HCP website (https://protocols.humanconnectome.org/HCP/7T/): " Each gradient table includes approximately 65 diffusion weighting directions plus 6 b=0 acquisitions interspersed throughout each run. Diffusion weighting consisted of 2 shells of b=1000 and 2000 s/mm2 interspersed with an approximately equal number of acquisitions on each shell within each run"

Preprocessing

Preprocessing software: The functional data used in this study is the preprocessed data from the Human connectome database. It has been preprocessed using the "Minimal Preprocessing Pipelines" of the Human Connectome Project. It uses FreeSurfer et FSL tools. More detail are available of the HCP website (https://www.humanconnectome.org/software/hcp-mr-pipelines) and the associated publication (Glasser et al. 2013, http://doi.org/10.1016/j.neuroimage.2013.04.127)

Normalization: As per the HCP pipelines (FLIRT, FNIRT)

Normalization template: MNI152 2mm isotropic

Noise and artifact removal: As per the HCP pipelines

Volume censoring: As per the HCP pipelines

Statistical modeling & inference

Model type and settings: General Linear Modelling, using Feat (from FSL). First level analysis using a high pass cutoff set at 200 sec., FILM prewhitening, and spatial smoothing of 4 mm FWHM for the original BOLD volumes (no smoothing for the functionnectome volumes). Second level analysis using mixed effects modelling (FLAME1).

Effect(s) tested: The effect tested depended on the tasks (various motor tasks, working memory task and language-semantic tasks). More details on the HCP website (https://protocols.humanconnectome.org/HCP/3T/task-fMRI-protocol-details.html)

Specify type of analysis: ☒ Whole brain ☐ ROI-based ☐ Both

Statistic type for inference (See Eklund et al. 2016): Voxel-wise inference

Correction: No correction

Models & analysis

n/a	Involved in the study
☒	☐ Functional and/or effective connectivity
☒	☐ Graph analysis
☒	☐ Multivariate modeling or predictive analysis
